# Nonlinear XUV signal generation probed by transient grating spectroscopy with attosecond pulses

**DOI:** 10.1038/s41467-019-09317-4

**Published:** 2019-03-27

**Authors:** Ashley P. Fidler, Seth J. Camp, Erika R. Warrick, Etienne Bloch, Hugo J. B. Marroux, Daniel M. Neumark, Kenneth J. Schafer, Mette B. Gaarde, Stephen R. Leone

**Affiliations:** 1 Chemical Sciences Division, Lawrence Berkeley National Laboratory, Berkeley, CA 94720 USA; 2 Department of Chemistry, University of California, Berkeley, Berkeley, CA 94720 USA; 3 Department of Physics and Astronomy, Louisiana State University, Baton Rouge, LA 70803 USA; 4 Department of Physics, University of California, Berkeley, Berkeley, CA 94720 USA

## Abstract

Nonlinear spectroscopies are utilized extensively for selective measurements of chemical dynamics in the optical, infrared, and radio-frequency regimes. The development of these techniques for extreme ultraviolet (XUV) light sources facilitates measurements of electronic dynamics on attosecond timescales. Here, we elucidate the temporal dynamics of nonlinear signal generation by utilizing a transient grating scheme with a subfemtosecond XUV pulse train and two few-cycle near-infrared pulses in atomic helium. Simultaneous detection of multiple diffraction orders reveals delays of ≥1.5 fs in higher-order XUV signal generation, which are reproduced theoretically by solving the coupled Maxwell–Schrödinger equations and with a phase grating model. The delays result in measurable order-dependent differences in the energies of transient light induced states. As nonlinear methods are extended into the attosecond regime, the observed higher-order signal generation delays will significantly impact and aid temporal and spectral measurements of dynamic processes.

## Abstract (web summary)

Ultrafast dynamics following light-matter interaction are governed by nonlinear processes. Here the authors show that initial nonlinear signal time-evolution is a consequence of phase grating accumulation using transient grating measurements with attosecond and near-infrared pulses.

## Introduction

The development of wave-mixing techniques in the extreme ultraviolet (XUV) and X-ray regimes represents the next frontier of nonlinear spectroscopy^1^. In optical^2^, infrared^3^, and radio-frequency^4^ nonlinear spectroscopies, highly selective multiphoton interactions are routinely employed to probe the structure and evolution of complex chemical systems dominated by rotational and vibrational dynamics^5^. Using femtosecond pulses, time-domain nonlinear techniques effectively reveal sub-picosecond transition states in chemical reactions^6^, mechanisms of energy transfer within photosynthetic complexes^7^, and the timescales of relaxation dynamics in semiconductors^8^. The extension of similar techniques to XUV/X-ray wavelengths and subfemtosecond pulse durations will provide critical insights into the fundamental dynamics associated with valence and core-level electronic transitions^9,10^.

High harmonic generation (HHG) and free electron laser (FEL)-based sources promise to exploit nonlinear processes to access ultrafast dynamics in the XUV. Transient grating schemes with intense 80 fs XUV pulses at FELs have successfully generated wave-mixing signals^11–15^. Similar schemes with XUV light produced by HHG paired with visible or near-infrared (NIR) pulses have probed acoustic and optical processes on timescales of tens to hundreds of femtoseconds^16,17^. Although table-top HHG can produce XUV pulses as short as 43 as^18^, low conversion efficiencies of 10^−6^ to 10^−8^ result in pulse energies insufficient to support nonlinear processes independently^19–23^. Nonetheless, pioneering experiments with subfemtosecond HHG XUV pulse trains and moderately intense femtosecond NIR pulses have isolated long-lived XUV four-wave-mixing signals through spectral filtering and noncollinear beam geometries. These methods have achieved background-free measurements of wavepacket dynamics in Rydberg and valence excited states of atomic^24,25^ and diatomic systems^26,27^. In conjunction with recent progress in subfemtosecond pulse generation at FELs^28,29^, these advances provide the foundation necessary for selective measurements on electronic timescales as well as for the direct probing of electronic correlations and strong-field effects in excited states. However, as these initial experiments focused primarily on the coupling of long-lived states, the impact of the nonlinear signal generation process itself has yet to be explored.

Here, we elucidate the temporal dynamics of nonlinear signal generation with broadband, ultrashort pulses. A beam geometry in which a NIR transient grating probes an XUV-induced coherence^30^ facilitates the simultaneous detection of up to five orders of resonance-enhanced nonlinear signals from 1s*np* Rydberg states in atomic helium between 20 and 24.6 eV. The time-domain characteristics of these signals reveal few-femtosecond time delays between the formation of distinct grating orders emitting at the energies of both the 1s*np* resonances and dressed states that appear in a strong infrared (IR) field, called light-induced states (LISs)^31–35^. Furthermore, we demonstrate that these delays lead to a less pronounced AC Stark shift of higher-order LIS features relative to lower-order features. Both the delays in emission and LIS energy shifts are directly reproduced by numerically solving the coupled Maxwell–Schrödinger equations for helium gas. We attribute the order-dependent delays to the accumulation of the AC Stark phase over the NIR pulse duration, increasing the modulation of a phase grating and thereby enhancing the efficiency of higher grating order formation with time. These results demonstrate a fundamental accumulation time inherent in the generation of nonlinear signals from electronically excited states, which represents an opportunity for increased selectivity and control in few-femtosecond and attosecond spectroscopy. Experimental observation of the fastest processes in nature, including electron correlation^36^ and transfer^37–39^, directly in the time domain is ultimately limited by the duration of the pulses utilized in these spectroscopic techniques. In the transient grating results described here, different transient grating orders emerge at different times within the duration of a few-cycle pulse and thus report on different time periods during ultrafast dynamics, providing a route towards measuring the timescales of dynamic processes occurring within the pulse duration. Furthermore, because of their distinct and predictable spectral characteristics, analysis of multiple grating orders may allow for the identification and isolation of desirable short-lived signals obfuscated in congested spectra. Utilization of this technique will therefore facilitate the observation and measurement of dynamics on electronic timescales in both the temporal and spectral domains.

## Results

### Transient XUV holography in atomic helium

**Fig. 1:**
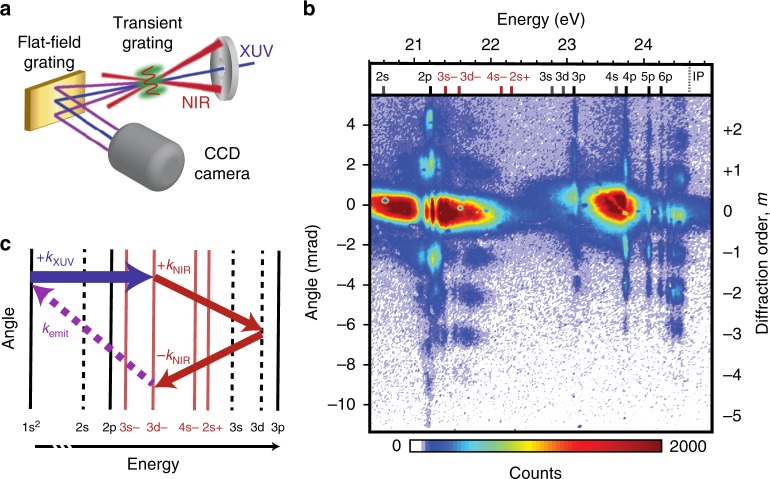
Experimental characterization and demonstration of extreme ultraviolet (XUV) transient grating spectroscopy. **a** Simplified experimental set-up depicting the formation of a transient grating in the helium sample (green) and the spectrometer employed to image the diffracted signals as a function of the delay between an subfemtosecond XUV pulse train and two few-cycle, time-coincident near-infrared (NIR) pulses. **b** A charge-coupled device (CCD) camera image taken 3.5 fs after pulse overlap. Positive delays indicate that the XUV pulse precedes the NIR grating. A number indicating the diffraction order, *m*, is provided to the right of the figure. Resonances and light-induced states (LISs) are assigned at the top of the image. **c** Energy level schematic depicting the three-photon pathway that emits at the energy of the LIS in the first grating order. Solid black lines correspond to *np* bright states, dashed lines correspond to *ns* or *nd* dark states, and solid red lines correspond to LISs

To generate wave-mixing emission, we utilize a transient grating geometry with a subfemtosecond XUV pulse train and two 6 fs NIR pulses centered at 800 nm (Fig. 1a, see Methods and Supplementary Note 1 for more details). The XUV pulse train initiates a coherent superposition of the ground state and the lowest-lying excited state manifold in gas-phase helium atoms at a density of ~ 3 × 10^17^ cm^−3^ in a 3 mm long cell. The time-coincident NIR pulses intersect the XUV at angles of approximately +1° and −0.75° to capitalize on the phase-matching conditions inherent in wave-mixing processes, producing spatially distinct higher-order grating signals. Multiple orders of nonlinear signals are imaged simultaneously as a function of XUV-NIR delay by a flat-field imaging spectrometer. Positive delays indicate that the XUV precedes the combined peak intensity of the NIR pulses. A charge-coupled device (CCD) camera image taken 3.5 fs after time overlap exhibits up to five distinct orders of resonance-enhanced nonlinear signals (Fig. 1b). In the zeroth order at a divergence angle of 0 mrad, depletion features provide clear evidence of XUV absorption by helium 1s*np* resonances. These long-lived states possess large absorption cross-sections^40^, facilitating the generation of multiple orders of nonlinear signals at the photon energies of the one-photon dipole-allowed (bright) *np* states. The slight distortion of the 2p state’s absorption and emission profiles is due to resonant pulse propagation effects^41,42^.

Notably, we also detect broad higher-order emission features at energies (e.g., ~21.8 eV) distinct from any *np* state. These features correspond to LISs, which, in a simple picture, can be described as intermediate dressed states in Raman-like two-photon transitions to one-photon dipole-forbidden (dark) states^31–35^. Enabled by strong coupling between a bright and dark state, LISs typically exist only when the XUV and NIR pulses overlap in time and space, and appear at energies approximately one NIR photon (~1.5 eV) above or below the associated dark state (Supplementary Note 2). The dominant LIS feature here originates from the 3d dark state located 1.5 eV above it in energy, and is referred to as the 3d− LIS (Fig. 1c, identification procedure in Supplementary Note 3)^33–35^. In a moderately intense NIR field, the 3d dark state and its LIS adiabatically blueshift in energy with the pulse envelope due to the AC Stark effect, as illustrated by the shift between the predicted and observed LIS energetic position in Fig. 1b. Importantly, LIS features are less blueshifted by ~ 0.1 eV with increasing order, indicating that the AC Stark effect is less pronounced for higher-order signals.

A time-domain holographic picture can be readily employed to conceptualize the observed spatial dependence of the nonlinear signals^30^. The intersection of the two noncollinear NIR beams causes spatial modulations in the electric field experienced by the helium gas, leading to periodically varying frequency-dependent changes in both the real and imaginary components of its refractive index. The spatial periodicity in the refractive index forms a grating that diffracts the XUV-induced free induction decay, the temporal characteristics of which map to the coherence time of the excited state^43^. According to the Bragg diffraction equation, the fringe spacing, *a*_NIR_, of this transient grating is given by $\lambda _{{\mathrm{NIR}}}{\mathrm{/}}\left( {{\mathrm{2}}\;{\mathrm{sin}}\left( {\theta {\mathrm{/2}}} \right)} \right)$, where *λ*_NIR_ is the NIR wavelength and *θ* is the crossing angle of the two NIR pulses. From this fringe spacing, it follows that each diffraction order, *m*, will appear at an angle defined by $m \cdot \lambda$_NIR_/*a*_NIR_. Alternatively, the diffracted signals can be described using the wavevector phase-matching requirements intrinsic to the perturbative interaction of one XUV photon and an even number of noncollinear NIR photons (additional details in Supplementary Note 4)^44^. However, as demonstrated in Supplementary Fig. 9, the NIR intensity dependence of the nonlinear signals described here is indicative of a nonperturbative regime in which distinct grating orders are composed of multiple orders of wave-mixing signal emitting at the same spatial location.

### Time- and energy-domain evolution of distinct grating orders

**Fig. 2:**
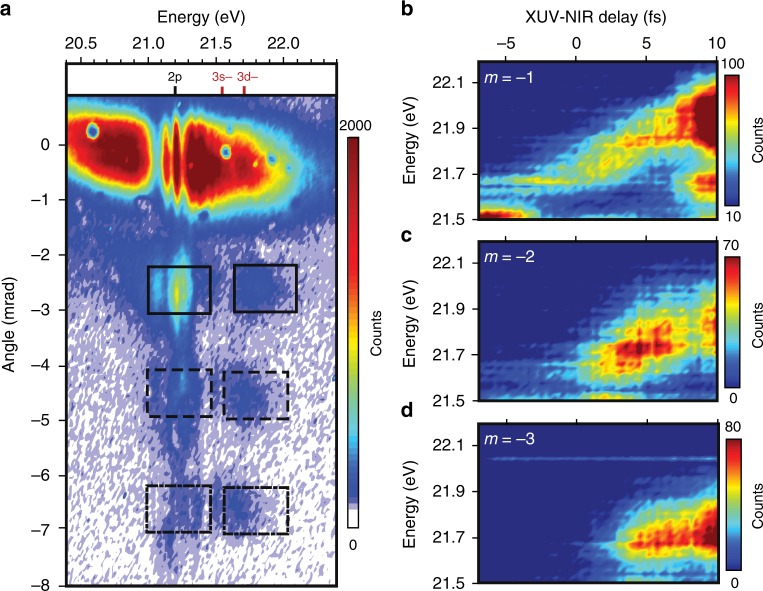
Order-dependent AC Stark shift of the 3d− light-induced state. **a** The experimental charge-coupled device (CCD) camera image taken 3.5 fs after overlap and cropped to emphasize the redshift of the light-induced state (LIS) spectra with grating order. The energy and delay dependence of the LIS emission features associated with the **b** *m* = −1, **c** *m* = −2, and **d** *m* = −3 grating orders are obtained by integrating vertically (0.7 mrad) over the three highest energy black boxes in **a**. The selected regions were chosen to avoid contamination from the distorted 2p state and allow for the full breadth of the energy shift

Figure 2 examines emission signals in different transient grating orders as a function of XUV-NIR delay, providing compelling insights into the origin of the order-dependent LIS energy shifts and, more generally, the temporal dynamics of nonlinear signal generation. In Fig. 2b–d, false color plots produced by integrating vertically over 0.7 mrad (10 pixels) for each of the features specified in Fig. 2a illustrate the evolution of LIS signals in both energy and time delay. The delay dependence of the features after pulse overlap (>8 fs) can be attributed to pulse reshaping in the dense He gas^45^, coupling to longer-lived states, and population accumulation in the dark state. These secondary considerations are detailed in Supplementary Note 5. In the three grating orders considered here, the LIS features initially appear centered around 21.66 ± 0.05 eV and subsequently broaden and blueshift in energy with increasing delay. A similar LIS shift has been documented extensively in collinear transient absorption geometries and is attributed to the AC Stark effect^34,35^. Interestingly, lower grating orders exhibit a greater relative energy shift than higher orders. The maximum of the first-order feature shifts more than 0.2 eV higher in energy (Fig. 2b), while the second- and third-order features shift only just over 0.1 eV (Fig. 2c) and 0.05 eV (Fig. 2d) respectively. Thus, LISs in higher grating orders experience a reduced AC Stark shift relative to those in lower orders. Another striking difference between grating orders is their delay dependence, with lower-order features emerging at earlier delays than higher-order features.

### Calculation of order-specific spectral and temporal profiles

**Fig. 3:**
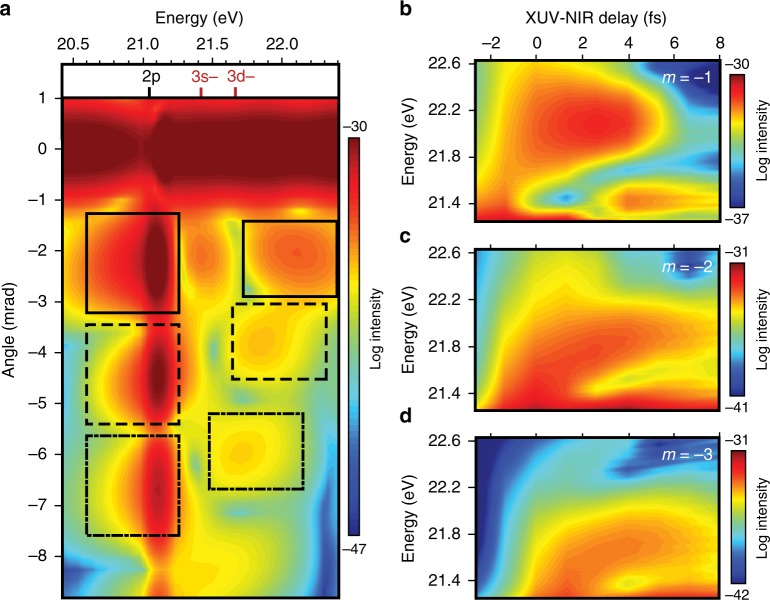
Far and near-field results of the time-dependent Schrödinger and Maxwell’s equations. **a** Calculated log spectral intensity for the 1s2p resonance and 3d− light-induced state (LIS) of helium gas propagated to the far field. The selected regions indicate distinct orders of either 2p or 3d− features. The delay dependence of the LIS emission features associated with the **b** *m* = −1, **c** *m* = −2, and **d** *m* = −3 grating orders are obtained by integrating vertically over the three highest energy black boxes in **a** in the near field. The data in **b**–**d** have been smoothed to reflect the lack of carrier envelope phase control in the experiment

To better understand the experimental observations, we calculated the observable spatio-spectral profile resulting from the transient grating interaction using the procedure detailed in ref. ^46^ (see Methods for more details). Using the single active electron (SAE) approximation, the coupled time-dependent Schrödinger equation (TDSE) and Maxwell’s equations were solved numerically using experimentally derived parameters. These calculations utilize a pseudopotential that accurately reproduces the energy levels for a singly excited He atom. The XUV pulse is assumed to be a single 330 as pulse centered at 23 eV, allowing for the simultaneous excitation of the entire manifold of 1s*np* states. Two identical 6 fs NIR pulses, each with a central frequency of 800 nm and an intensity of 2 × 10^12^ W cm^−2^, are crossed at angles of +1° and −0.75° with respect to the XUV beam to replicate the noncollinear experimental geometry. To accommodate the resulting non-cylindrical symmetry, the response is calculated in one transverse direction only^47^. These calculations yield the space- and time-dependent electric field at the end of a thin helium gas jet, ignoring the resonant pulse propagation effects observed experimentally for the 2p state. Figure 3a shows the spatio-spectral intensity profile after transforming to the far field for an XUV-NIR delay of 4 fs. The profile is plotted on a log scale to highlight weak higher-order features. The delay and energy dependence of the LIS features designated in the higher energy windows of Fig. 3a is shown in Fig. 3b–d. The calculation successfully reproduces the multiple diffraction orders visible at the 2p and LIS energies as well as the order-dependent variations in energy and delay for the LIS features observed experimentally.

**Fig. 4:**
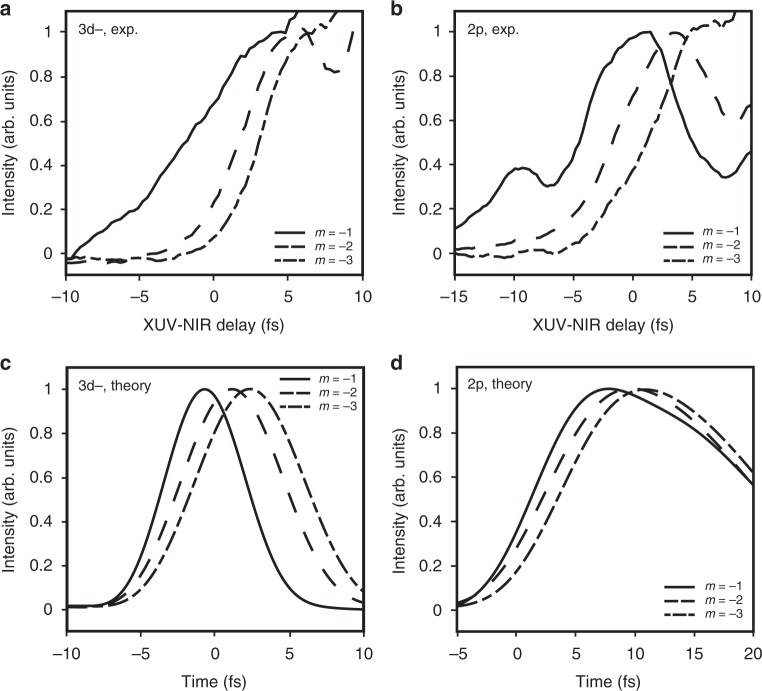
Experimental and calculated delay in the emergence of higher-order signals. **a** Experimental extreme ultraviolet and near-infrared pulse delay dependence of the lowest three nonlinear grating orders of the 3d− light-induced state (LIS) and of **b** the 2p state obtained by integrating over the higher (21.6–22.1 eV; 0.7 mrad) and lower energy windows (21.0–21.4 eV; 0.7 mrad), respectively, in Fig. 2a. **c** Real-time dependence calculated for distinct orders of 3d− LIS features obtained by integrating over the higher energy windows in Fig. 3a and of **d** 2p features obtained by integrating over the lower energy windows after transforming the results into the nearfield. The decay of the 2p signals is due to a decoherence lifetime of 20 optical cycles added to the time-dependent Schrödinger equation calculation to simulate the experimentally observed coherence time

To further quantify the delay dependence of different grating orders and characterize the dynamics of nonlinear signal generation, the experimental LIS features indicated in Fig. 2b–d are integrated over an energy bandwidth of 0.5 eV (21.6–22.1 eV). Integration areas were chosen to minimize spurious effects from nearby states and to accommodate the energy shift of the features. The resulting delay dependence demonstrates that while the temporal profiles associated with the LISs in each grating order are similar, higher orders indeed emerge at later XUV-NIR delays (Fig. 4a). The second and third diffraction order LIS features peak at 1.5 ± 0.6 fs and 2.4 ± 0.6 fs, respectively, after the first order. To verify that these delays are not unique to the transient LISs, the same analysis was performed for the long-lived 2p state (Fig. 4b). Integrating over 0.3 eV (21.1–21.4 eV) results in measured delays of 1.8 ± 0.8 fs between first and second orders and 3.6 ± 0.8 fs between the first and third orders. The local maximum at −10 fs may be due to propagation effects. The delays observed for distinct grating orders of long-lived *np* states are consistent with those for the transient 3d− LIS. Additional examples are provided in Supplementary Note 6.

In the calculations, we access the evolution of distinct diffraction orders as a function of real time, rather than XUV-NIR delay, by transforming the windows shown in Fig. 3a into the time domain and the near field. Time zero corresponds to the peak of the NIR pulses, which arrive 4 fs after the initial XUV excitation. For the 3d− LIS, calculated delays of 1.8 fs between first and second orders and 3.0 fs between the first and third orders compare well to the experimental results (Fig. 4c). Similar delays of 2.1 fs and 3.3 fs are observed for the 2p state (Fig. 4d). The comparison between delay and real time is particularly appropriate for the earliest delays when the time-integrated emission signals are not yet dominated by cumulative effects. As shown in Supplementary Note 7, the calculated delay dependence agrees with Fig. 4. The agreement between the delay-dependent experimental and time-dependent theoretical results suggests that the experimentally observed delays between diffraction orders originate in real-time differences in the temporal dynamics of higher-order signal generation: different diffraction orders are formed at different times during the nonlinear interaction.

### AC Stark phase grating model

**Fig. 5:**
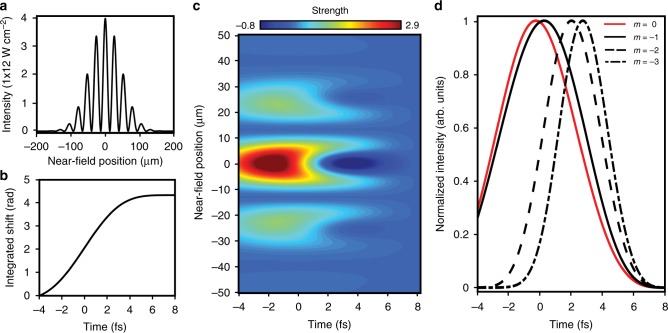
Model of AC Stark phase grating accumulation and nonlinear signal generation. **a** Spatially modulated intensity profile generated by two crossed near-infrared (NIR) pulses. **b** The calculated NIR-induced AC stark shift plotted as a function of real time, where time zero corresponds to the time at which the extreme ultraviolet (XUV) pulse interacts with the system and the peak of the NIR pulse. **c** A false color plot shows the amplitude of the dipole moment modulated by both the AC Stark phase grating and an amplitude grating in the nearfield as a function of time. Later times exhibit an increased modulation depth. **d** In the far field, different grating orders are plotted as a function of time during the NIR pulse

To better understand the origin of the time delay between the emergence of different grating orders in the Rydberg states and LISs, we compare the results with a simple model based on the formation of a transient phase grating due to the crossed NIR pulses. In a noncollinear geometry, the crossing of the NIR beams results in an interference pattern in the NIR intensity (Fig. 5a) that generates both a spatial grating in the AC Stark phase and an amplitude grating in the XUV-excited population. The time- and space-dependent dipole, *d*(*x*,*t*), of an excited atom oscillating at XUV frequency, *ω*, that interacts with perturbing NIR pulses can be expressed as:

1 $$d\left( {x,t} \right)\,{\mathrm{ = }}\,f\left( {x,t} \right){\mathrm{cos}}^2(k_{{\mathrm{NIR}}}x)e^{{i}\phi (x,t)}e^{ - i\omega t}{\mathrm{ + }}\,{\mathrm{c}}{\mathrm{.c}}{\mathrm{.,}}\;{\mathrm{for}}\;t \ge t_0.$$

Here, *f*(*x,t*) describes the envelopes of the XUV field in space and the NIR field in time, and ${\mathrm{cos}}^2\left( {k_{{\mathrm{NIR}}}x} \right)$ represents an amplitude grating where $2k_{{\mathrm{NIR}}} = 2\pi /a_{{\mathrm{NIR}}}$ is the wavevector associated with the NIR intensity grating. The NIR-induced phase shift, $\phi (x,t)$, can be expressed as:

2 $$\phi {(x,t) = }{\int _{t_0}^{t}} \frac{{\Delta {E}\left( {x,t \prime } \right)}}{\hbar }{{\mathrm{d}}t}\prime,$$

where $\Delta E\left( {x,t\prime } \right)$ is the AC Stark shift of the excited state energy and *t*_0_ is the arrival time of the XUV pulse. This phase shift accumulates over the course of the NIR pulse (Fig. 5b) and can therefore be approximated by the relationship:

3 $$\phi \left( {x,t} \right) \approx \Delta \left( {x,t} \right){\mathrm{cos}}^2\left( {k_{{\mathrm{NIR}}}x} \right),$$

where $\Delta (x,t)$ is the phase shift due to the NIR envelope and is modulated by the sinusoidal grating pattern.

This combined phase and amplitude grating leads to diffraction of the near-field XUV electric field into multiple orders in the far field. The diffracted XUV field is proportional to the dipole moment. Figure 5c demonstrates that the dipole becomes more strongly modulated with time due to the accumulation of the AC Stark phase, meaning that the efficiency with which higher-order diffraction signals are generated will increase with time. This evolution can be quantified by sampling the near-field dipole moment at specific points in real time and transforming it into the far field, thereby providing a view into grating order formation (Fig. 5d). At early times, only the zeroth and first orders are generated due to the population grating. However, at later times, as higher-order modulations appear in the dipole moment because of the accumulating phase grating, higher diffraction order peaks arise in the far field. The excellent agreement of this simple model’s time dependence with both the delay-dependent experiment and the full calculations suggests that the observed delays in higher-order signal generation arise due to the time associated with the build-up of the AC Stark phase over the NIR pulse and validates the use of a phase grating model as a physical picture for nonlinear signal generation in this experimental configuration. Contrary to a perturbative picture in which the highest order signals peak at pulse overlap^48^, the highest order signals here are delayed relative to the peak of the NIR pulse (0 fs). The grating orders’ intensity dependence also must be described as nonperturbative and can be explained via the phase grating model. Supplementary Note 8 provides additional details about both the phase grating model and its nonperturbative characteristics.

Finally, the phase grating model also provides insight into the attenuation of the LISs’ AC Stark shift with increasing grating order. As shown in Fig. 5d, higher-order signals arise only after accumulation of the AC Stark phase. Thus, features in higher diffraction orders emerge later in the NIR pulse and subsequently have less time to accumulate AC Stark phase after generation. Since the AC Stark phase originates from the energy shift of the state (Eq. 2), these higher-order features will therefore exhibit a less pronounced energy shift relative to lower-order features that interact with the NIR pulse over a longer timeframe. This effect is only evident for the transient LIS features because their emission exists only when the NIR pulse is present, whereas the dipole moment from the comparatively long-lived *np* states persists well after the NIR-induced Stark shift has passed.

## Discussion

In summary, the few-femtosecond temporal dynamics of nonlinear signal generation is investigated in gas-phase helium using a transient grating geometry between two noncollinear few-cycle NIR pulses and a subfemtosecond XUV pulse train. Simultaneous measurements of multiple diffraction orders of nonlinear signals reveal significant delays in the emergence of higher-order signals associated with both Rydberg states and LISs. Calculations using the coupled Maxwell–Schrödinger equations in the SAE approximation reproduce these delays and demonstrate that they originate in real-time differences in signal generation between distinct grating orders. Furthermore, because higher grating orders arise later within the NIR pulse as a consequence of this delay, higher-order LIS features exhibit a less prominent AC Stark shift than those in lower orders. Finally, we introduce a conceptual model to relate the delay times to the accumulation of an AC Stark phase grating over the course of the NIR pulse, explicitly defining the observed delays as a fundamental property of nonlinear signal generation. Given the intense interest in developing nonlinear spectroscopies in the HHG and FEL communities, these delays will be important to the design and interpretation of nonlinear experiments that probe subfemtosecond dynamics. Experimentally obtainable pulse durations can obfuscate the temporal signatures of short-lived processes, limiting the dynamics that can be studied in the time domain. This issue is amplified in nonlinear spectroscopic techniques requiring noncollinear beam geometries that degrade the time resolution dictated by the pulse duration. These transient grating results provide a compelling alternative. The knowledge that different grating orders emerge at different times in the NIR pulse can be utilized systematically to unravel dynamics occurring within the NIR pulse duration. In the spectral domain, the grating order-dependent energy shift of short-lived features suggests a mechanism by which features associated with ultrafast processes can be discriminated from spectra with overlapping and complex features. This work represents one of the potentially many experiments in which these intrinsic delays will impact the behavior of nonlinear signals.

## Methods

### Experimental scheme

The experimental apparatus employed for these measurements shown as Supplementary Fig. 1 has been described previously^27^. Briefly, 22 fs NIR pulses produced by a 1 kHz, 2 mJ commercial laser system (Femtopower HE, Femtolasers) are spectrally broadened in a neon-filled hollow core fiber with an inner diameter of 400 µm and subsequently compressed by 6 pairs of broadband chirped mirrors (Ultrafast Innovations) to produce 6 fs pulses with a spectral bandwidth extending between 550 and 950 nm. The pulses transmitted through a 50:50 beamsplitter are focused by 50 cm focal length mirror into the vacuum chamber (10^−6^ Torr) containing a sample cell with xenon gas to produce a pulse train of 2–3 subfemtosecond pulses in the XUV via high harmonic generation. A 0.2 µm Sn filter (Lebow, 17–24 eV transmission) spectrally filters the XUV and co-propagating NIR light to include only the 13th and15th harmonics. A gold-plated toroidal mirror focuses the XUV through an annular mirror into a second 3 mm long cell containing the helium gas target at densities of approximately 3 × 10^17^ cm^−3^. The XUV intensity is estimated to be between 10^8^ and 10^10^ W cm^−2^.

The NIR pulses reflected from the 50:50 beamsplitter before high harmonic generation are delayed relative to the XUV by a piezoelectric stage and then divided into two arms by a second 50:50 beamsplitter. The reflected and transmitted beams are displaced above and below the hole of the annular mirror in the XUV beam path such that they are recombined with the XUV in the target cell at angles of approximately 1° and 0.75° respectively and focused to roughly 100 µm FWHM (full width at half maximum) spots with an intensity of 2 × 10^12^ W cm^−2^. The delay between these two NIR arms is controlled by a second stage positioned in the transmitted beam path. For these experiments, the position of the second stage is set to ensure the NIR arms are time-coincident. Temporal and spatial overlap of the pulses is determined by the appearance of fringes on a CCD camera positioned at the focus due to the interference between the NIR beam used for HHG and the noncollinear probe beams.

After propagating through 3 mm of approximately 3 × 10^17^ cm^−3^ of helium gas in the target cell, a 0.2 µm Al filter (Lebow, 20–76 eV transmission) removes any residual NIR light. The transmitted XUV spectrum is dispersed by a flat-field grating (01–0464, Hitachi) and recorded by an x-ray CCD camera (Pixis XO 400B, Princeton Instruments). A step size of 300 as was chosen for the delay between the XUV and noncollinear NIR pulses in order to resolve time dynamics on the few-femtosecond timescale. At each delay, 1500 laser pulses were accumulated three times to obtain an appropriate signal-to-noise ratio. The delay at which the transient 2s+ zeroth grating order feature at 22.1 eV obtains its peak absorbance is assigned a value of 0 fs (Supplementary Fig. 4). This feature was chosen to avoid contamination from 1s2p pulse propagation effects. The energy axis of the CCD camera was calibrated daily using atomic transition line data available from the National Institute of Standards and Technology (NIST). Camera image data, false color plots, and lineouts of higher-order signals are presented in terms of raw counts (flux) on the CCD camera.

### Calculated spatio-spectral profile and time dependence

The coupled TDSE and the Maxwell wave equation are solved numerically in the SAE approximation to generate spatio-spectral profiles in the far field. First, an initial frequency-domain electric field is defined at the beginning of a helium jet by specifying each input field as a focused Gaussian beam. The time-domain transform of this field provides a two-color source term for TDSE, which results in a space- and time-dependent dipole moment that can be transformed back into the frequency domain. The calculations proceed by space-marching the frequency-domain driving and the generated fields in the propagation (*z*) direction. At each point in *z*, we transform the total electric field to the time domain and calculate the space- and time-dependent polarization field (proportional to the time-dependent dipole moment) by solving the TDSE at a number of different points across the (transverse, *x*) laser profile. The polarization field is then transformed to the frequency domain and used as the source term in propagating to the next *z*-plane. At the end of the medium we transform the resulting space- and frequency-dependent electric field to the far field. The two NIR beams both have Gaussian transverse profiles with waists of 64 µm, and the XUV beam waist is 22 µm. Although a train of two to three attosecond pulses was employed experimentally, the use of single XUV pulse in the calculations is justified given the relative timescales of the pulse train and the NIR pulses. The density of the He gas is 10^19^ cm^−3^, and we work in the thin-medium limit and propagate through only 0.01 mm of gas, corresponding to only space-marching one step in the forward direction. This means that we incorporate all aspects of the self-consistent interactions that lead to absorption of the light propagating along the axis, and the emission of the light propagating (diffracting) in off-axis directions, but we ignore effects such as resonant pulse propagation that occur in longer/denser media. In this limit, our medium is equivalent to a 1 mm long gas with a density of 10^17^ cm^−3^, almost an order of magnitude lower than the estimated experimental gas density. A lower gas density was chosen for the calculations to demonstrate that the delays observed between nonlinear grating orders in the experimental data do not originate from resonant propagation effects. An increase in gas density is not expected to significantly modify the conclusions drawn here.

## Supplementary information

Supplementary Information

## Data Availability

The data generated during and analyzed during the current study are available from the corresponding author on reasonable request.
