# Supplementary material for: Nonlinear XUV signal generation probed by transient grating spectroscopy with attosecond pulses
Source: Nat Commun. 2019 Mar 27;10:1384. doi: 10.1038/s41467-019-09317-4 (PMC6437156; doi:10.1038/s41467-019-09317-4)
Supplement: Supplementary file 1 — Supplementary Information [file 41467_2019_9317_MOESM1_ESM.pdf]

Supplementary Information

Nonlinear XUV Signal Generation Probed by Transient Grating Spectroscopy with Attosecond Pulses

Fidler *et al*.

Supplementary Note 1: Pulse Overlap Determination

The experimental apparatus is described in detail in the Methods and in ref. 1. A schematic showing the generation of XUV light and the implementation of the time delay between the XUV and NIR pulses is provided here as Supplementary Fig. 1. To ensure synchronization of the NIR pulses, we arranged the two piezo-stages in the interferometer in series. The first stage is positioned before the NIR arm is split into two by the second beamsplitter in order to control the delay of both the upper and lower beams simultaneously. Fine adjustments to the second stage in the beam path transmitted through the beamsplitter compensate for daily differences in alignment of the two NIR arms.

A commercial dispersion scan device (d-scan, Sphere Ultrafast Photonics) is utilized to measure the NIR spectrum and optimize its pulse duration before entering the vacuum chamber. The results of one such measurement are given in Supplementary Fig. 2. The NIR bandwidth extends from 550 – 950 nm, which supports a near-transform limited pulse duration of 5 fs. While the duration of the pulses in the vacuum chamber will be somewhat lengthened compared to those measured with the d-scan because they do not propagate through equivalent amounts of air, experimental risetimes indicate pulse durations of approximately 6 fs.

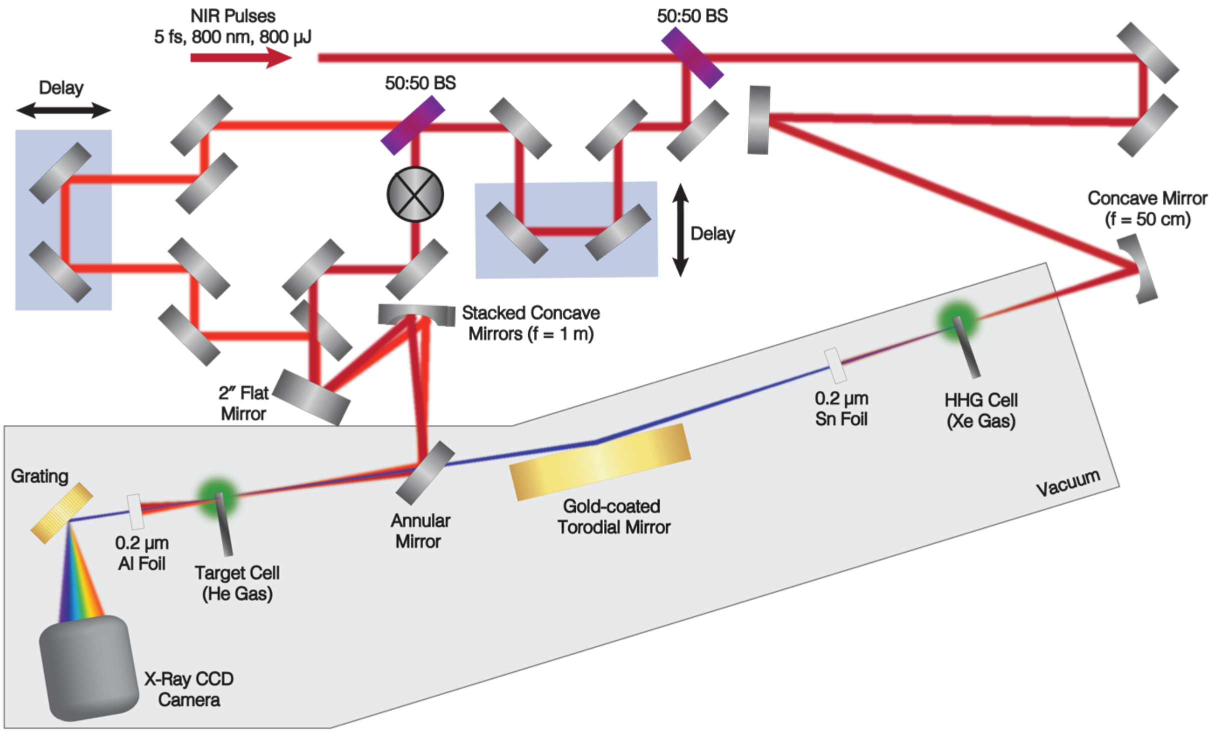

Supplementary Fig. 1 | Experimental nonlinear attosecond spectroscopy apparatus

Three independently controlled beam paths allow for spatial isolation of signals that originate from different interaction pathways. BS = beamsplitter, f = focal length, Sn = tin, Al = aluminum.

The quality of spatial and temporal overlap between the three pulses is assessed using a CMOS camera (DCC1545M, Thorlabs) as shown in Supplementary Fig. 3. In order to obtain an observable signal from the XUV arm on the camera, the Sn filter is removed to allow the driving NIR pulses to propagate with the XUV. A removable pick-off mirror inserted into the beam path after the annular mirror directs all three NIR beams to a camera placed at the focus outside of vacuum. Because of the arrangement of the two stages, overlap is first found between the HHG arm and the upper NIR beam. After positioning the focal spots of the two noncollinear beams on

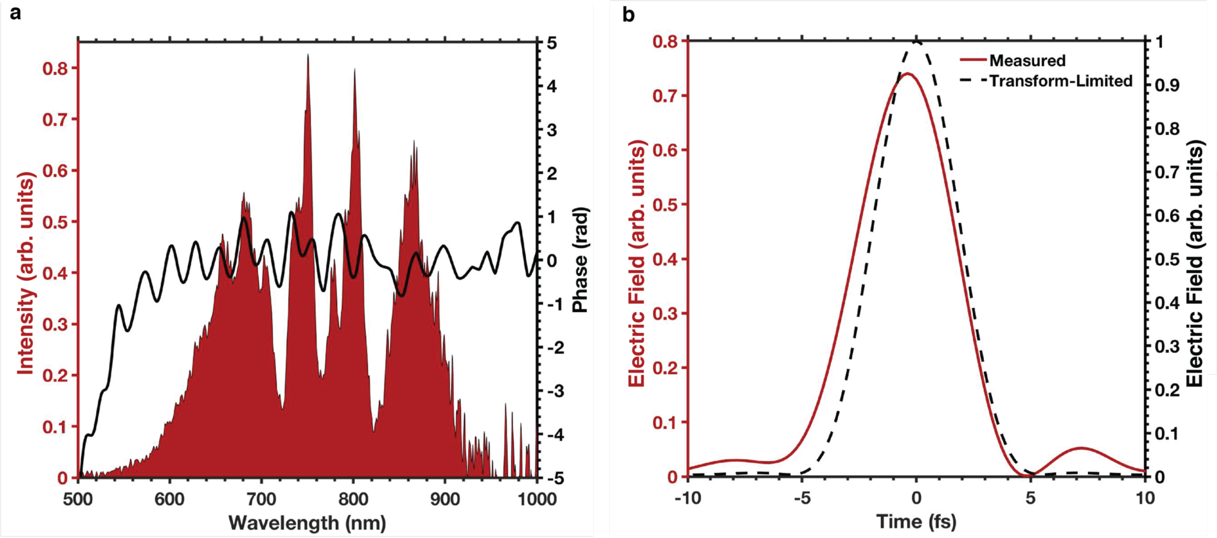

Supplementary Fig. 2 | NIR pulse characterization

a, The spectrum of the NIR pulse (red) and its phase (black). **b,** A pulse duration of 5 fs is obtained from a dispersion-scan measurement.

top of each other (spatial overlap), the first delay stage is moved manually until fringes appear on the overlapping beam profiles (Supplementary Fig. 3a). The fringes indicate that the lengths of the two beam paths are equal and thus the pulses are overlapped in time. Overlap between the HHG arm and the lower NIR beam is then established by modifying the position of the second stage. In addition to indicating time overlap, the fringes reveal the crossing angle, θ_{crossing}, between the XUV and each of the NIR pulses. Using the equation:

$$\theta_{\text{crossing}} = 2\sin^{-1}\left(\frac{\lambda_{\text{NIR}}}{2x}\right)$$

where λ_{NIR} is the central NIR wavelength and x is the fringe spacing, the crossing angles are determined to be 1.0º and 0.75º for the upper and lower arms respectively.

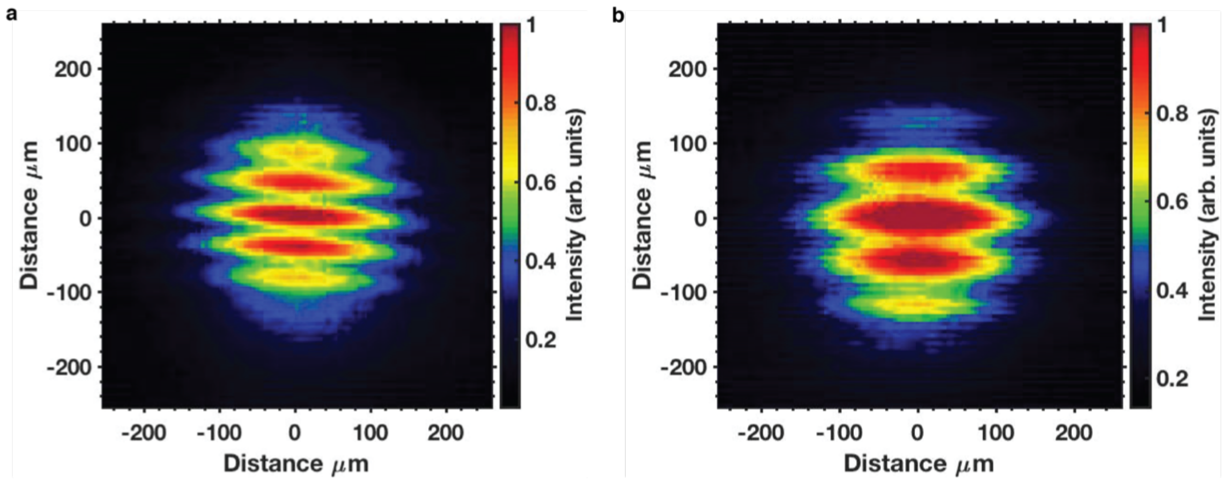

Supplementary Fig. 3 | Evaluation of Spatial and Temporal Overlap

a, Overlap of the upper arm and the NIR light used for HHG results in fringes in the beams' spatial profile. The crossing angle is determined to be 1.0º. **b,** The fringe spacing at overlap of the HHG and lower arms is larger than that in **(a)**, leading to a crossing angle of 0.75º.

Supplementary Note 2: Zeroth Grating Order Signals

The zeroth grating order in these noncollinear wave-mixing experiments essentially constitutes a transient absorption measurement. In Supplementary Fig. 4a, absorption features from the 2p state and two LISs are observed. As discussed in the helium transient absorption literature[2,3], the exceptionally large cross section of the 1s2p resonance allows for significant resonant pulse propagation effects in a collinear geometry. Propagation effects are dependent upon the susceptibility and dephasing time of a transition, the frequency of light used for the excitation, and target gas density and propagation length. In brief, the XUV-induced oscillating dipole generates an electric field out of phase with the driving field. As this dipole-induced electric field propagates through the medium, it too will excite atomic resonances, which in turn produce radiation out of phase with the initial dipole-induced electric field. This process results in the formation of secondary pulses with durations dependent on the gas density and propagation

length. Meanwhile, the NIR pulse imposes a phase shift on the XUV-induced electric field so that it is no longer completely out of phase with driving field. When the timescale of the first subpulse is comparable to that of the IR perturbation, the interplay of these two effects results in additional spectral features, like those observed in the zeroth order of the 2p state. The lack of additional features in the higher lying *np* states can be attributed to their weaker response to the field.

The two LIS features can be assigned using reported literature energy values to the 3d- (21.7 eV) and the 2s+ (22.1 eV) LISs. As discussed in the manuscript, LISs are intermediate dressed states in a two-photon Raman-like transition to a dark state. Some examples are given in Supplementary Fig. 4b. These transient signals were employed in these experiments to identify the exact position of time overlap. In the CMOS-based method described in the previous section, the duration of the pulses used to determine overlap is lengthened by the addition of a fused silica window and additional air to the beam path. While increasing the pulse duration ensures that the fringes are easier to find manually, time overlap is consequently only approximate. Time overlap is more precisely determined via the time dependence of a transient absorption feature in a simple gas phase system. We examine the rise of 2s+ LIS to establish the exact delay at which time overlap is observed (Supplementary Fig. 4c). The 2s+ transient state was chosen over the stronger 3d- state for this measurement because it is located farther away from the 2p state in energy and is therefore less impacted by prominent NIR-induced sidebands and potential propagation effects. However, it is important to note that it is technically difficult to determine time overlap exactly in these experiments. Unlike in ref. 4, the lack of carrier envelope phase stabilization in our experiment precludes the observation of fast subcycle (4ω or higher)

oscillations in the zeroth order. Furthermore, as discussed in Supplementary Note 4, each grating order does not correspond to unique wave mixing order, meaning that the observed grating orders are not equivalent to these subcycle oscillations.

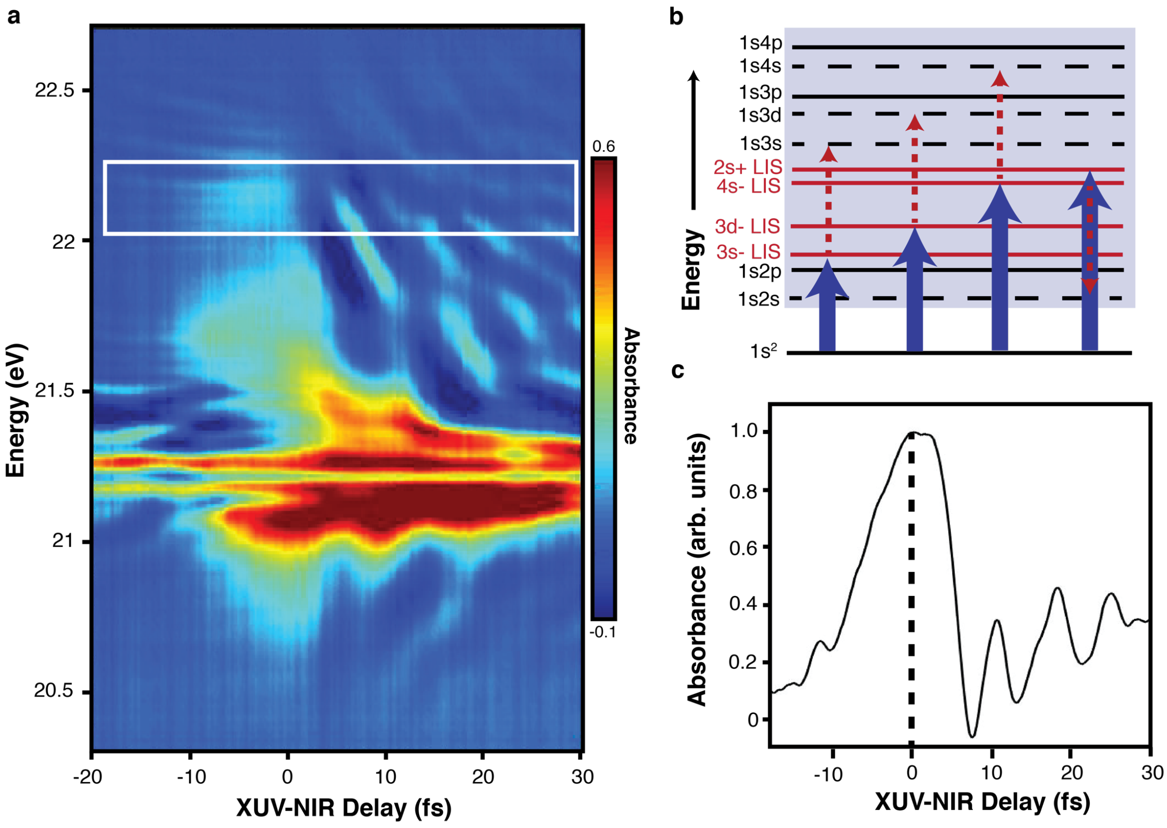

Supplementary Fig. 4 | Zeroth Grating Order Time Dependence

a, A false color plot showing the absorbance of the 2p state in the zeroth grating order as a function of energy and XUV-NIR delay. Splitting and additional spectral components are apparent in the long-lived 2p state (21.2 eV). Some of these spectral components appear to extend into the feature associated with the 3d- LIS around 21.7 eV. The 2s+ LIS (22.1 eV, white box) is also observed in the zeroth order. **b,** Energy level schematic depicts the two-photon Raman-like pathways that result in LISs. Solid black lines correspond to *np* bright states, dashed lines correspond to *ns* or *nd* dark states, and solid red lines correspond to LISs. **c,** The 2s+ LIS in the zeroth grating order is used to determine the delay at which the pulses are overlapped in time. The oscillations beginning at ~9 fs are due to ringing from the NIR-induced truncation of the 2p state's free induction decay (FID)[5]. The dotted line indicates the delay defined as time overlap.

Supplementary Note 3: Identification of Light Induced States

To assign the broad emission features between 21.6 and 22.2 eV to the appropriate LIS, we employed a joint experimental-theoretical approach.

Polarization Measurements

Dipole selection rules dictate that while both *ns*± and *nd*± LIS features appear when the polarization of the XUV and NIR pulses are parallel ($\theta = 0°$), only the *nd*± LIS features remain in a perpendicular polarization ($\theta = 90°$)[3]. We introduced a half-wave plate into the noncollinear NIR arm to modify its polarization relative to XUV in order to determine whether the LIS features observed in higher grating orders are associated with an *ns* or an *nd* dark state. As shown in Supplementary Fig. 5, the primary nonlinear LIS feature can be observed in both the perpendicular and parallel polarization geometries. Note that the presence of the wave plate increases the pulse duration and decreases the intensity of the signals, making higher order signals more difficult to achieve. To quantify the maintenance of the m = -1 LIS feature between both polarizations, the ratio between the baseline-subtracted first order LIS (*nl*-) and zeroth order 2s+ LIS can be compared. In the parallel polarization, both LISs are present and the *nl*-/2s+ ratio is 0.34. In the perpendicular polarization, the first order *nl*- LIS is maintained but the zeroth order 2s+ LIS disappears, resulting in an increase of the *nl*-/2s-+ ratio by a factor of 14.8 to 5.03. As the feature between 21.6 and 22.2 eV is maintained in both polarizations, selection rules dictate that it must be assigned to an *nd*± LIS. Of the *nd*± LISs accessible in this experiment, the expected energy of the 3d- LIS (21.6 eV) best correlates with the observed feature, especially given that a shift to higher energies at later delays is expected due to the AC Stark shift.

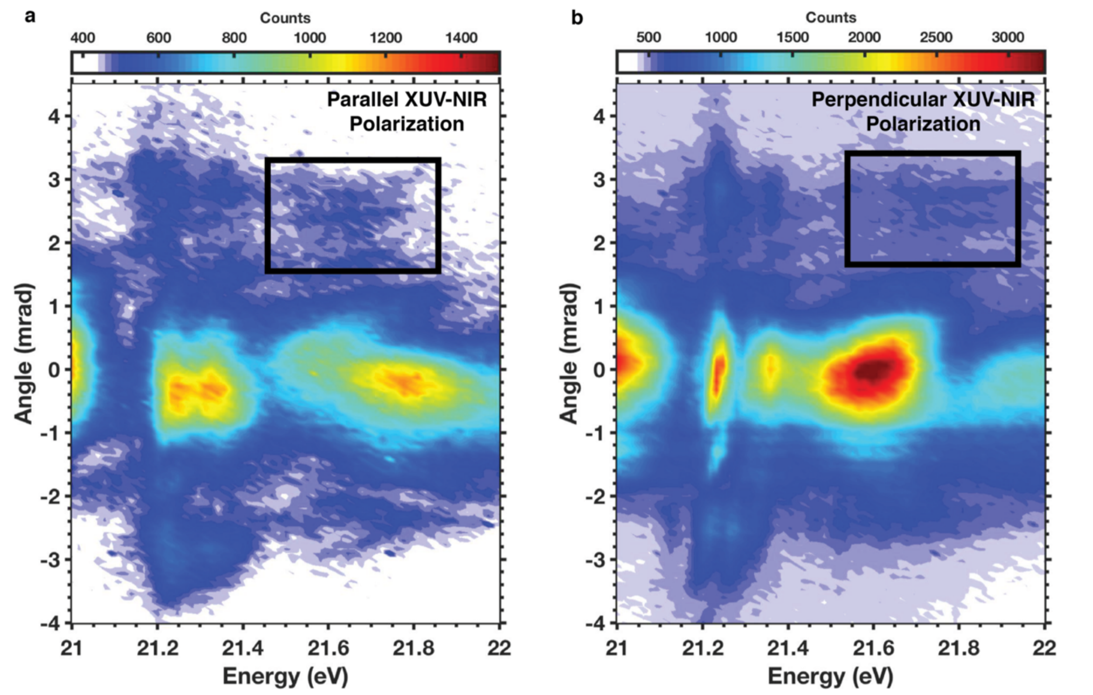

Supplementary Fig. 5 | The LIS feature is maintained in both parallel and perpendicular XUV-NIR polarizations

CCD camera images cropped to focus on the 2p resonance and the primary LIS for **a,** parallel and **b,** perpendicular polarizations between the XUV and NIR pulses. The black boxes indicate the position of the LIS.

Calculated Spatio-Spectral Profiles

This assignment can be verified further theoretically. As described in the methods section, the coupled time dependent Schrödinger equation (TDSE) and the Maxwell wave equation are solved numerically in the single active electron (SAE) approximation to generate spatio-spectral profiles in the far field. The effect of resonant pulse propagation effects in these wave-mixing experiments are therefore not considered here, but will be the focus of future studies. Looking specifically at the energy landscape surrounding the 2p resonance, multiple broad features distinct from the *np* states appear prominently in the calculated profile (Supplementary Fig. 6). Note that the calculations shown below have been done using slightly longer NIR pulse durations

(12 fs) in order to improve the spectral resolution and more easily identify different features. Changing the central wavelength of the NIR pulses employed in the calculation shifts the position of the broad features, as one would expect for LISs. Assuming a LIS picture, the features will shift toward their parent dark state with increasing wavelength. The feature that best corresponds with the most prominent experimental LIS shifts toward higher energies with increasing wavelength, indicating that its associated dark state is located higher in energy than the feature. Furthermore, removal of the *nd* dark states from the calculation substantially diminishes the intensity of this LIS feature, indicating that it must be associated with a *nd* dark state located ~1.5 eV away (Supplementary Fig. 7). The most likely dark state candidate is the 3d state. Therefore, these results support the assertion that these features originate from the 3d- LIS.

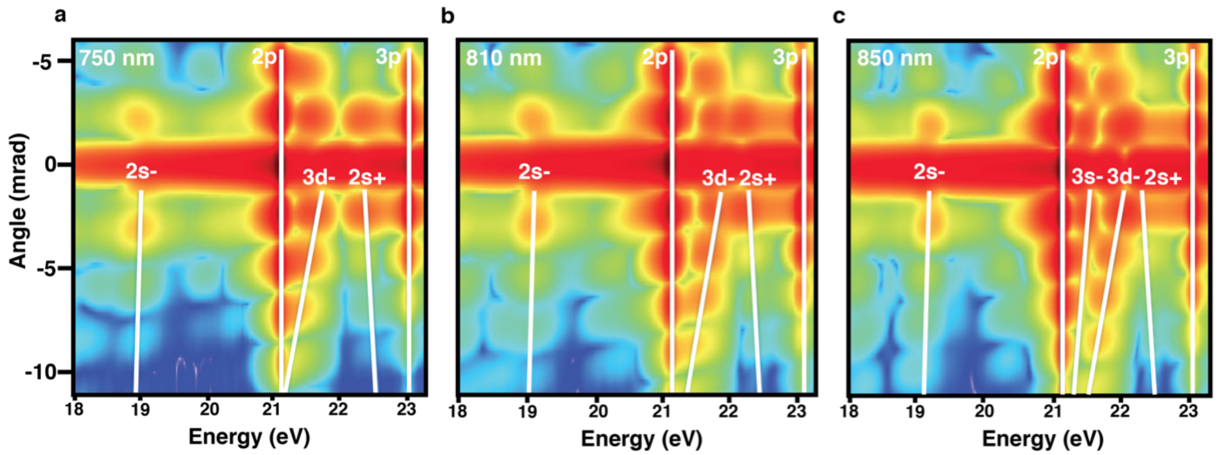

Supplementary Fig. 6 | Wavelength dependence of LIS features

Calculated spatio-spectral profile in the far field for NIR pulses with a central wavelength of **a,** 750 nm, **b,** 810 nm, and **c,** 850 nm. The while lines draw attention to features of interest. The assignment of each feature is given at the top of each line.

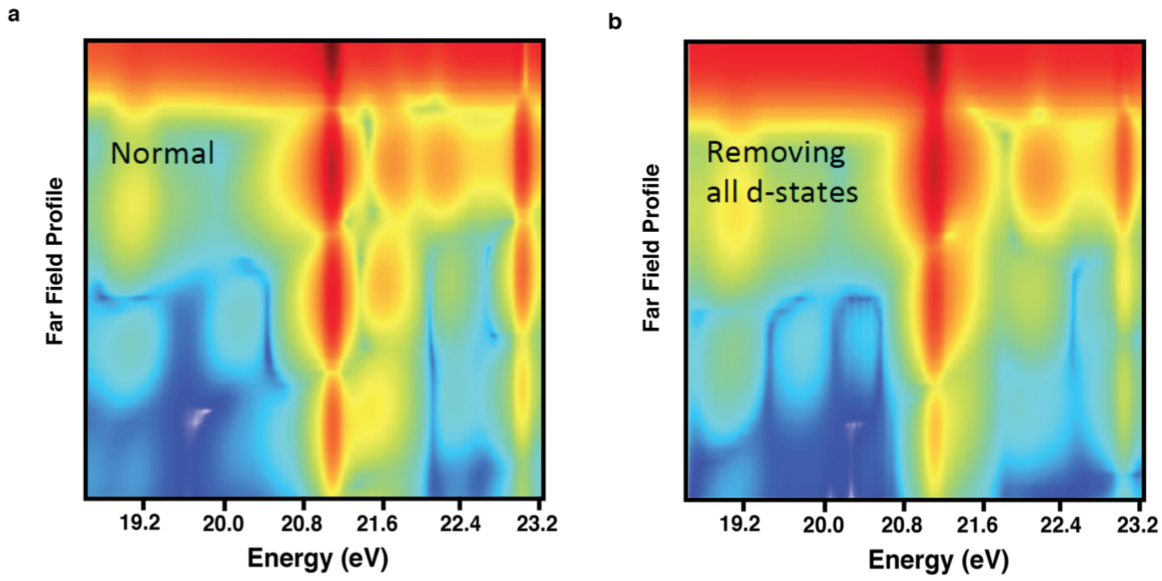

Supplementary Fig. 7 | LIS features with and without *nd* dark states

Calculated spatio-spectral profile in the far field using an 800 nm central wavelength. The region surrounding the 2p state is selected to compare the results of the calculations **a**, with and **b**, without the one photon dipole forbidden *nd* states.

Supplementary Note 4: Wave-Mixing Pathways

In a holographic picture, the generation of nonlinear signals result from diffraction of an input beam off of a sinusoidal grating. Deviations from a perfect sinusoid produce higher order nonlinear signals[6]. In a complementary picture, spatially-isolated nonlinear signals can be intuitively described by wavevector phase-matching requirements intrinsic to the perturbative interaction of one XUV photon and even numbers of noncollinear NIR photons[7]. These interactions generate a macroscopic polarization with significant contributions from higher-order terms:

$$P = \varepsilon_0(\chi^{(1)}E + \chi^{(2)}EE + \chi^{(3)}EEE + \ldots)$$

where ε_0 is the vacuum permittivity, $\chi^{(n)}$ is the nth-order susceptibility, and E is the electric field of the excitation. In an isotropic medium, these nonlinear terms result in emission from processes

dependent upon odd orders of the nonlinear susceptibility. Myriad different pathways can emit at a given energy due to the broad bandwidth of the ultrashort pulses utilized in this experiment. The broadband XUV pulse generates a coherent superposition of multiple *np* excited states, which is then probed by the time-coincident NIR pulses. Beating in the time dependent signal occurs when two or more NIR photons couple multiple states excited in the initial interaction to the same final state. If both of the photons originate from same NIR beam, then the resulting wave-mixing signal is produced via a ladder-type coupling pathway in which the NIR photons couple the two states through a dark state positioned between them in energy (Supplementary Fig. 8a). Signals resulting from this type of wave mixing process are discussed extensively in ref. 8–10. For systems in which the desired initial and final excited states are not separated by two photons, V- (Λ-) coupling pathways offer an alternative. In these pathways, two final states are coupled via a dark state that lies below (above) them. To isolate the emission of these pathways from the linear response, two noncollinear photons are required (ref. 1, Supplementary Fig. 8b). Significant evidence of the coupling of multiple states to the same feature can be observed in the data presented here. The 1.5 fs oscillations in the time dependence of the 4p state shown below in Supplementary Fig. 11a can be tied to the interference of a V-type pathway in which the 4p couples back to itself and a ladder type pathway involving the 2p state (Supplementary Fig. 8c). Furthermore, as discussed further in the following section, the persistence of the 3d- LIS features after time overlap in the first, second, and third grating orders can be explained by a 3d dark state-mediated Λ-type coupling of features associated with the strong 2p state to the LIS.

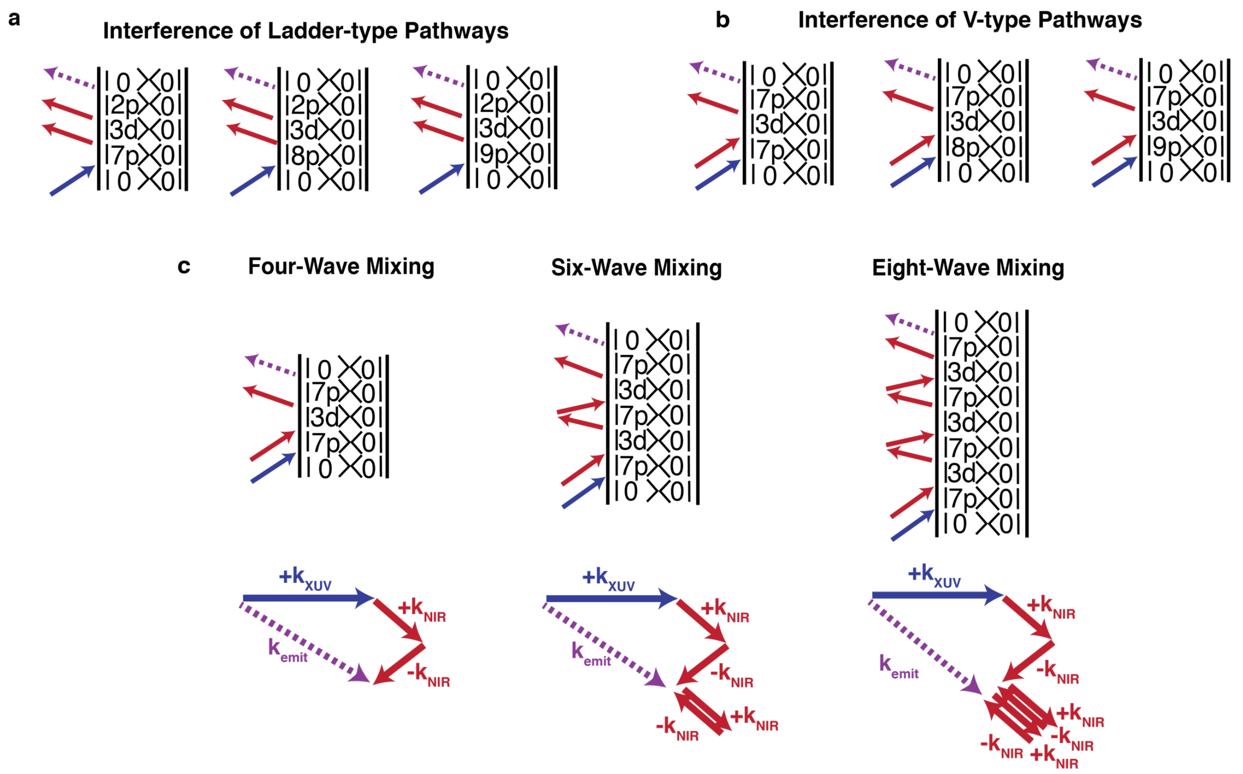

Supplementary Fig. 8 | Emission features originate from a variety of different coupling pathways

a, Feynman diagrams showing that multiple higher energy *np* states can couple through the 3d dark state to the lower-lying 2p state via a ladder-type coupling pathway. **b,** The same manifold of higher energy *np* states can couple back to themselves via a V-type coupling pathway. **c,** Interference of ladder and V-type pathways leads to half-cycle beating in the 4p state. These pathways may not be perfectly resonant. **d,** While higher order wave mixing pathways can emit at larger divergence angles than the lower order pathways presented here, multiple orders of wave mixing signals can also emit at the same angle. Blue arrow = XUV, red arrow = NIR, Dotted purple arrow = wave mixing emission.

Finally, it is important to note that due to the variety of coupling pathways allowed in a noncollinear geometry like the one employed here, the individual grating diffraction orders do not necessarily correspond to a single order in the nonlinear susceptibility. Hermann *et al.* [4] demonstrates significant contributions from five photon pathways ($\chi^{(5)}$) through 4ω oscillations measured directly in the time domain. As demonstrated in Supplementary Fig. 8d, higher-order

wave-mixing processes can be phase matched to emit at the same spatial location as lower order processes, resulting in a nonperturbative effects. To verify that the experiment exhibits features consistent with a nonperturbative regime, the NIR intensity dependence of individual grating orders can be measured experimentally. According to ref. 8, the flux (counts) of a pure four-wave mixing process should scale quadratically with NIR intensity in a perturbative picture. To smoothly modulate the NIR intensity, a waveplate and polarizer were installed into the NIR beampath and two orders of nonlinear transient grating signal were obtained. As in Supplementary Fig. 5, the addition of these optics increases the pulse duration and thus decreases the peak intensity somewhat in the interaction region. At each intensity point, a static spectrum with and without He gas is taken and the power in the NIR beampath is recorded. In Supplementary Fig. 9, it is apparent that the first grating order signal does not scale quadratically with the measured NIR power. The data is best fit by a power function, indicating that it cannot be described perturbatively. Multiple orders of wave-mixing signals at the same spatial location has substantive impacts on the intensity of individual grating orders and contribute nonperturbative scaling observed in the experimental data, both within and between final emission states[6].

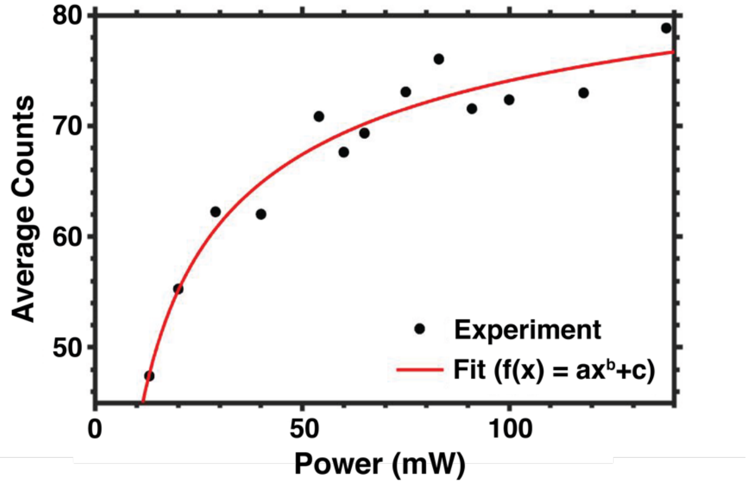

Supplementary Fig. 9 | Dependence of first grating order signal on NIR power

The average number of counts obtained by integrating over the first grating order LIS signal as a function of the intensity (power) of the NIR arm increases rapidly at lower powers and begins to plateau at higher powers. The experimental data is best fit with a power function (a = -133.7, b = -0.4101, c = 94.31).

Supplementary Note 5: Time Dependence of Light Induced States

The higher grating order features observed at the energetic position of the 3d- LIS persist for up to 50 fs after time overlap (Supplementary Fig. 10a). As described in references 11–15, LIS features typically only exist when the condition of temporal and spatial overlap between the XUV and NIR pulses is satisfied. There are several potential explanations for the experimentally observed LIS behavior.

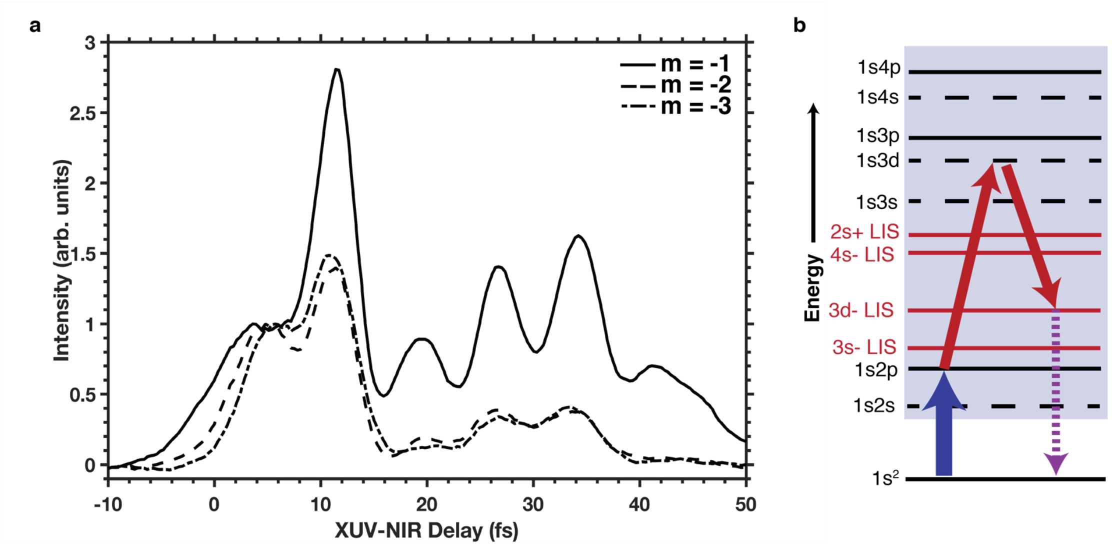

Supplementary Fig. 10 | Delay-dependence of the 3d- LIS features

a, Experimental plots for the lowest three nonlinear grating orders of the 3d- LIS (21.6 - 22.1 eV) using the same parameters as Fig. 3 demonstrating persistence of the features well past time overlap. **b,** An energy diagram showing the likely pathway leading to coupling of the 2p state to the 3d- LIS. The NIR photons (red) may be of different energies (lengths) because of the broad bandwidth of the pulse. Blue arrow = XUV, red arrow = NIR, Dotted purple arrow = wave-mixing emission.

Dark State Population

An alternative explanation is that the LIS itself actually persists after overlap due to the accumulation of population in the 3d dark state. In ref. 11, the persistence of the LIS is shown to be a function of the detuning between the bright and dark state. When highly detuned, no net population builds-up in the dark state, leading to LISs that disappear once the XUV is no longer present. This case has been documented extensively in the transient absorption literature. However, as the states become less detuned, the dark state does have the opportunity to accumulate some real population, allowing for emission after overlap. The 5 fs NIR pulses

employed in these experiments may support a large enough bandwidth to satisfy the condition for population accumulation. More work is needed to validate this consideration.

Wave-Mixing Coupling Mechanisms

Although the off-axis features appear at the energetic position of a particular state, as demonstrated in Supplementary Note 4, they originate from multiphoton interactions and therefore may contain contributions from multiple states. Therefore, while the energy profile of a feature may correspond to the 3d- LIS, its time dependence may be characteristic of the coherence time of one of the longer-lived *np* bright states. Of these bright states, the most likely candidate on the basis of cross section and the NIR bandwidth is the 2p state. Either XUV-induced dipole emission (free induction decay) or secondary electric fields induced by propagation effects (see Supplementary Note 2) can couple to the 3d- LIS through the 3d dark state (Supplementary Fig. 10b). The spacing of oscillations observed in the delay dependence resembles the strong hyperbolic side bands due the NIR-induced truncation of the 2p dipole observed on-axis[5], perhaps indicating that these features can take part in wave-mixing processes if the cross section of their parent state is above some threshold value.

Pulse Propagation Effects

The propagation of a pulse through a dense medium with strong resonances has already been discussed within the context of the 2p state. However, as shown in ref. 16, the effects of an optically thick resonant medium on pulse propagation need not be confined to the 2p state. As the pulse propagates through the medium, the helium gas will attenuate resonant frequencies and shape the spectral phase of nonresonant frequencies. The coherent manipulation of the spectral

phase can modify the time-dependent transient population and therefore the observed dynamics of the probed state. While the higher grating order LIS features are spatially separated from the XUV pulses, their time dynamics are strongly influenced by the exciting pulse. Furthermore, the wave-mixing emission itself is resonant with the LIS and therefore could be subject to reshaping.

Supplementary Note 6: Additional Examples of Temporal Dynamics of Nonlinear Signal Generation

As mentioned in the main text of the manuscript, delays in nonlinear signal generation can be observed in other states besides those shown in Fig. 4. Two additional examples are provided in Supplementary Fig. 11. Delays in nonlinear signal generation are apparent in the lowest three grating orders of the 4p state as well (Supplementary Fig. 11a). At the photon energy of the 4p resonance, the second order feature emerges 3 ± 1 fs after the first order signal. A more pronounced delay of 4 ± 1 fs is measured between the first and third orders. Due to its lower cross section, the helium 4p state should be minimally affected by propagation effects that may affect the 2p state. As discussed in Supplementary Fig. 8, the fast modulations are due to the interference of ladder and V-type pathways.

In addition, to verify that these delays can be observed in experimental systems other than helium, we examined the near-threshold ($4p^5(^2P_{1/2})ns/nd$) autoionizing states of atomic krypton between 14.0 and 14.6 eV (Supplementary Fig. 11b). A step size of 300 as was chosen for the delay between the XUV and noncollinear NIR pulses. At each delay, 1500 laser pulses were accumulated three times to obtain an appropriate signal to noise ratio. A delay of 1.8 ± 0.4 fs is measured between the first and second grating orders at the energy of the $4p^5(^2P_{1/2})8s/6d$ state.

Unfortunately, only two grating orders beyond the zeroth order can be obtained in these experiments as the cross sections of krypton's autoionizing states are an order of magnitude smaller than helium's and the XUV flux at the target is reduced significantly in the 14 eV energy range due to poor transmission through the necessary 0.1 μm indium foils (Lebow, 11-17 eV transmission). Furthermore, resonant pulse propagation effects have never been observed in these states, lending credence to our claim that these effects are not the origin of the delay.

It is important to note the integration areas leading to these plots must be carefully selected for each order, particularly for transient states that shift dramatically in energy and for features that lie close to other states in either space or energy. For example, in these experiments, the higher order 3d- LIS features shift in energy by as much as 0.3 eV over the NIR pulse duration. Phase matching requirements dictate that the divergence angle will change as well since the energy of the photons involved in the wave-mixing process are modified due to this shift. The window therefore must take into account both the shift in energy and in angle. Furthermore, the 3d- LIS lies in a congested portion of the spectra due to the nearby 2p resonance, which is spectrally distorted due to pulse propagation effects, and other LISs above and below it in energy. To obtain an accurate delay, the window should be constructed to avoid effects from these other states. In the case of the data presented here, the window integrated over for the 3d- LIS first order feature was narrowed in energy by 0.1 eV relative to the second and third orders to avoid contamination from the nearby 3s- LIS. When measured at the peak of the rise of the feature, the delay remains the same regardless of energy window, but the broader window leads to a distorted initial rise time.

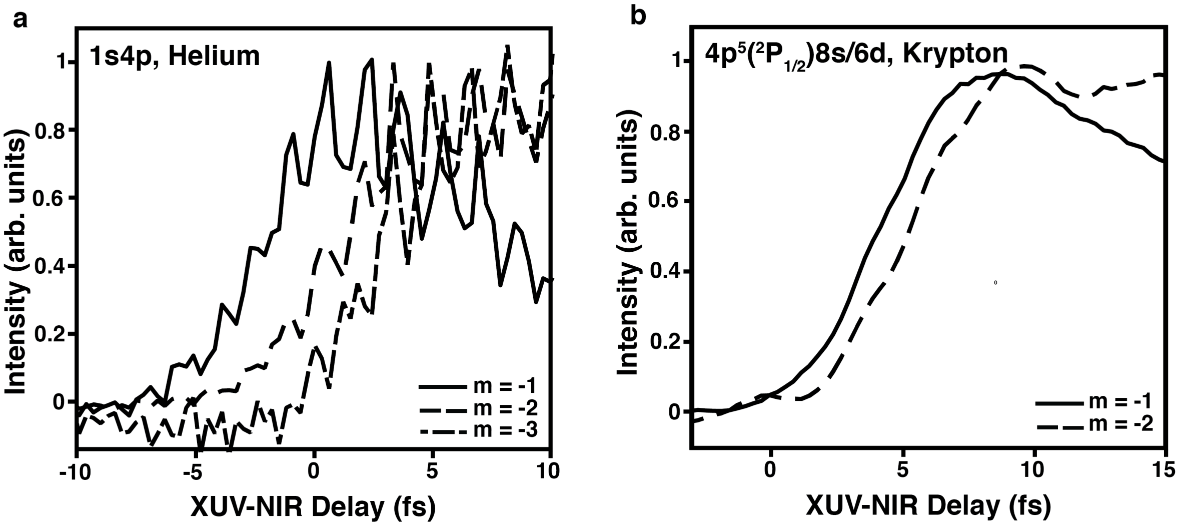

Supplementary Fig. 11 | Additional experimental examples of delays in nonlinear signal generation

a, Experimental plots for the lowest three nonlinear grating orders of helium's 4p state (23.74 - 23.81 eV) and **b,** krypton's $4p^5(^2P_{1/2})8s/6d$ state (14.09 – 14.12 eV).

Supplementary Note 7: Delay Dependent Calculations

Main text Figs. 4c-d show the calculated emergence of the lowest three nonlinear grating orders (m = -1, -2, -3) as a function of real time, rather than the experimental parameter, XUV-NIR delay. The experimental delay and calculated time plots will appear most similar at small delays because there is less integrated history between the XUV and NIR pulses at smaller delays than at larger ones. To directly compare to experiment, the emergence of the grating orders was calculated as a function of XUV-NIR delay (Supplementary Fig. 12). Like the real time-dependent plots in main text Figs. 4c-d, the XUV-NIR delay-dependent plots demonstrate that the first order features emerge before the second order features, which then arise before the third order. These delay-dependent features exhibit subcycle oscillations due to interference of

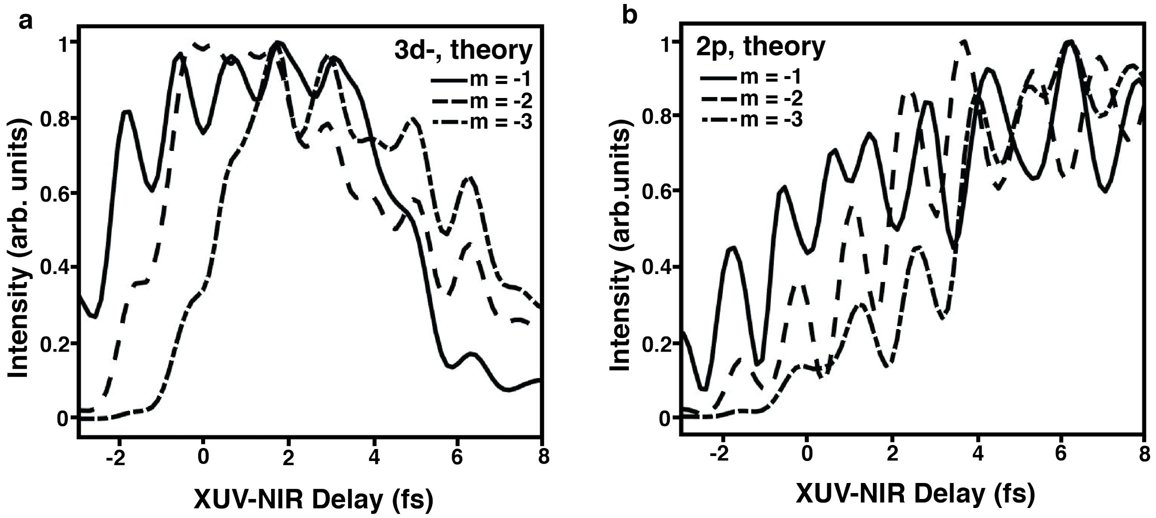

Supplementary Fig. 12 | XUV-NIR delay dependent calculations for the emergence of higher order signals **a,** Delay dependence calculated for the lowest three grating orders of the 3d- LIS obtained by integrating over the higher energy windows in Fig. 3. **b,** Delay dependence of the lowest three grating orders of the 2p state obtained by integrating over the lower energy windows in Fig. 3. The data is not smoothed to show the subcycle oscillations.

pathways that differ by two photons. The subcycle features are not observed in the experimental data because the experiment was not carrier envelope phase (CEP) stabilized.

Supplementary Note 8: AC Stark Phase Grating Model

AC Stark Phase Grating

In the phase grating model, the space- and time-dependent quantity $\Delta(x,t)$ represents the phase shift accumulated at the maximum combined NIR intensity (i.e. at the peak of each intensity fringe) as it varies across the NIR focus. The AC Stark shift at a particular location, $\Delta E(x,t)$, is approximated by the ponderomotive energy corresponding to the intensity at that point. This leads to the smooth increase of $\Delta(x,t)$ with time as shown in main text Fig. 5b. The envelope

function $f(x,t)$ used in main text Fig. 5c (and to calculate the data in main text Fig. 5d) matches the spatial profile of the XUV and time-dependence of the NIR, and thus approximately models the LIS, which only exist in the presence of the NIR field.

The time-dependence of the different diffraction orders shown in main text Fig. 5d is calculated by doing a far-field transform of the instantaneous amplitude of the (complex) amplitude of the positive frequency component of the field in main text Eq. (4) at different times during the NIR pulse. Fig. 5d then plots the spatially separated, normalized yields of the different diffraction orders in the far field. Although the model includes, to first order, the contributions of an amplitude grating in the dipole moment due to the amplitude of the polarization field being modulated directly by the NIR grating, the time dependence of model originates primarily from the accumulation of the phase grating. For Supplementary Fig. 13, the contribution of the constant amplitude grating is removed, revealing the time dependence associated with a pure AC Stark phase grating. Note that the effect of the XUV envelope is also ignored in these calculations. As in main text Fig. 5c, the modulation depth of the dipole moment increases with time in Supplementary Fig. 13a. In the far field, again higher order transient grating signals emerge later than lower order signals (Supplementary Fig. 13b). However, without the contribution from the amplitude grating, the first order signal shifts away from the zeroth order signal in time, decreasing the delay between the first and second orders from 1.73 fs to 1.4 fs. Slight asymmetries in the NIR angles relative to the XUV do not substantially impact these results.

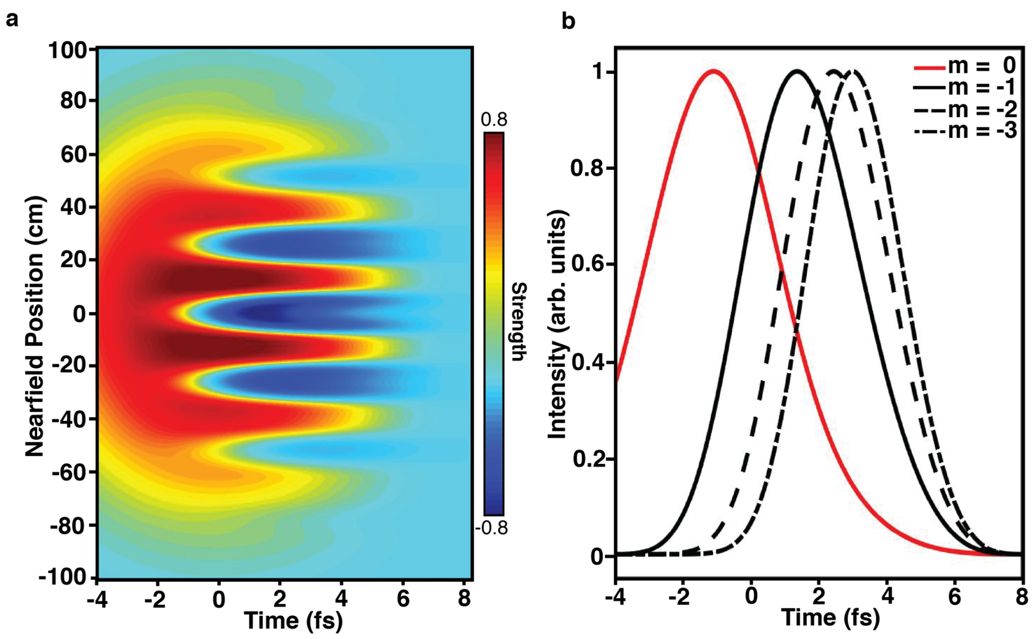

Supplementary Fig. 13 | XUV-NIR delay dependent calculations for the emergence of higher order signals

a, A false color plot shows the amplitude of the dipole moment modulated by a pure AC Stark phase grating in the nearfield as a function of time. **b,** Different grating orders are plotted as a function of time during the NIR pulse in the far field.

Intensity Dependence

In addition to replicating the time delay between different orders observed in the experimental data and the full calculation, the combined phase and amplitude grating model also predicts that the strength of the features will not scale perturbatively with NIR intensity, as illustrated in Supplementary Fig. 14. This nonperturbative scaling can be understood by expanding the spatially-dependent term for the phase grating as a series of Bessel functions:

$$e^{i\Delta\cos(k_{\mathrm{NIR}}x)} = \sum_{m=-\infty}^{\infty} i^m J_m(\Delta)\, e^{imk_{NIR}x}$$

where Δ is the phase shift at a particular intensity in an explicitly noncollinear geometry, and k is the wavevector associated with the NIR phase grating. This expansion is valid for any value of Δ.

First, the initial peak of the Bessel function of order m shifts to larger Δ as m increases, meaning that the higher diffraction orders appear later than the lower orders as the accumulated phase shift increases with time. Second, the Bessel functions introduce an oscillatory behavior as a function of the accumulated phase shift. Therefore, the features associated with each grating order will saturate as the intensity increases beyond a certain threshold and decrease.

This finding provides deeper insight into the strength of the grating orders in the experimental data as it explains why higher order features appear as strong as or even stronger than lower order ones under certain conditions.

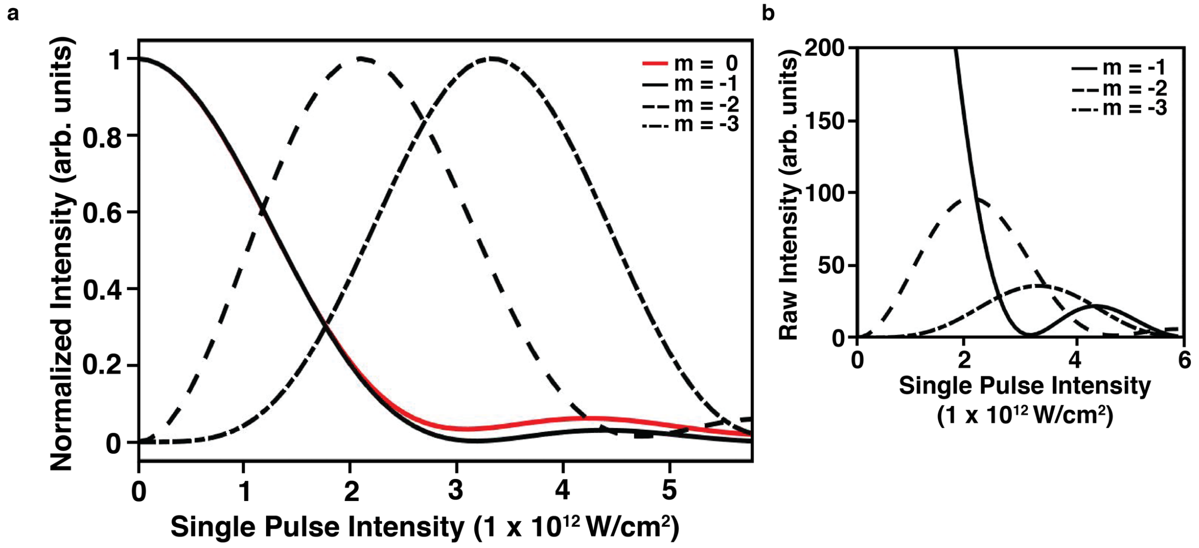

Supplementary Fig. 14 | Nonperturbative intensity dependence in the AC Stark phase grating model

a, The intensities of the lowest four (m = 0, 1, 2, and 3) grating orders saturate and decrease with increasing NIR intensity. The lineouts are normalized to allow all orders to be viewed simultaneously. **b**, Without normalization, the relative intensity of the lowest three nonlinear grating orders (m = 1, 2, and 3) at a particular NIR intensity can be compared. The intensity of the m = 1 order is amplified by the contribution of a first order amplitude grating.

Supplemental References

1. Warrick, E. R. *et al.* Multiple pulse coherent dynamics and wave packet control of the N 2 a" 1 Σ g + dark state by attosecond four wave mixing. *Faraday Discuss.* **212,** 157–174 (2018).
2. Bell, M. J., Beck, a. R., Mashiko, H., Neumark, D. M. & Leone, S. R. Intensity dependence of light-induced states in transient absorption of laser-dressed helium measured with isolated attosecond pulses. *J. Mod. Opt.* **60,** 1506–1516 (2013).
3. Reduzzi, M. *et al.* Polarization control of absorption of virtual dressed states in helium. *Phys. Rev. A - At. Mol. Opt. Phys.* **92,** 033498 (2015).
4. Herrmann, J. *et al.* Multiphoton transitions for robust delay-zero calibration in attosecond transient absorption. *Springer Proc. Phys.* **162,** 83–86 (2015).
5. Beck, A. R., Neumark, D. M. & Leone, S. R. Probing ultrafast dynamics with attosecond transient absorption. *Chem. Phys. Lett.* **624,** 119–130 (2015).
6. Blouin, A., Denariez Roberge, M.-M. & Galarneau, P. Degenerate n-wave mixing in a saturable absorbers. *J. Opt. Soc. Am. B Opt. Phys.* **8,** 578–583 (1991).
7. Mukamel, S. *Principles of Nonlinear Optical Spectroscopy*. (Oxford University Press, 1995).
8. Cao, W., Warrick, E. R., Fidler, A., Leone, S. R. & Neumark, D. M. Near-resonant four-wave mixing of attosecond extreme-ultraviolet pulses with near-infrared pulses in neon : Detection of electronic coherences. *Phys. Rev. A* **94,** 021802 (2016).
9. Cao, W., Warrick, E. R., Fidler, A., Neumark, D. M. & Leone, S. R. Noncollinear wave mixing of attosecond XUV and few-cycle optical laser pulses in gas-phase atoms: Toward multidimensional spectroscopy involving XUV excitations. *Phys. Rev. A - At. Mol. Opt.*

Phys. **94,** 053846 (2016).

10. Cao, W., Warrick, E. R., Fidler, A., Leone, S. R. & Neumark, D. M. Excited-state vibronic wave-packet dynamics in H2 probed by XUV transient four-wave mixing. *Phys. Rev. A* **97,** 023401 (2018).
11. Wu, M., Chen, S., Camp, S., Schafer, K. J. & Gaarde, M. B. Theory of strong-field attosecond transient absorption. *J. Phys. B At. Mol. Opt. Phys.* **49,** 062003 (2016).
12. Chini, M. *et al.* Sub-cycle oscillations in virtual states brought to light. *Sci. Rep.* **3,** 1105 (2013).
13. Chan, W. F., Cooper, G. & Brion, C. E. Absolute optical oscillator strengths of the electronic excitaiton of atoms at high resolution: Experiemental methods and measurements for helium. *Phys. Rev. A At. Mol. Opt. Phys.* **44,** 186–204 (1991).
14. Pfeiffer, A. N. *et al.* Alternating absorption features during attosecond-pulse propagation in a laser-controlled gaseous medium. *Phys. Rev. A - At. Mol. Opt. Phys.* **88,** 051402(R) (2013).
15. Liao, C. T., Sandhu, A., Camp, S., Schafer, K. J. & Gaarde, M. B. Beyond the Single-Atom Response in Absorption Line Shapes: Probing a Dense, Laser-Dressed Helium Gas with Attosecond Pulse Trains. *Phys. Rev. Lett.* **114,** 143002 (2015).
16. Strasser, D. *et al.* Coherent interaction of femtosecond extreme-uv light with He atoms. *Phys. Rev. A - At. Mol. Opt. Phys.* **73,** 021805(R) (2006).
